# Potentiometric Performance of a Highly Flexible-Shaped Trifunctional Sensor Based on ZnO/V_2_O_5_ Microrods

**DOI:** 10.3390/s21072559

**Published:** 2021-04-06

**Authors:** Alfred Bekoe Appiagyei, Jeong In Han

**Affiliations:** Department of Chemical and Biochemical Engineering, Dongguk University, Seoul 04620, Korea; 2018126719@dgu.ac.kr

**Keywords:** flexible sensor, PET fiber, simultaneous hydrothermal, multifunctional detectors, bending, wearables

## Abstract

A trifunctional flexible sensor was fabricated on a polyethylene terephthalate (PET) fiber surface. Synthesized ZnO and ZnO/V_2_O_5_ composite were coated on ZnO seed layer sputtered PET fiber. X-ray diffraction (XRD) and photoelectron spectroscopy (XPS) techniques confirmed the exact formation of ZnO and ZnO/V_2_O_5_. The fabricated ZnO/V_2_O_5_ on ZnO seeds base temperature sensor recorded better electrical properties and reversibility with a maximum temperature coefficient resistance (TCR) of 0.0111 °C^−1^. A calibration curve (R = 0.9941) within glucose concentration of (10 µM–10 mM) was obtained at +0.8 V vs. Ag/AgCl from current-voltage curves which assisted in calculating glucose sensitivity, limit of detection (LOD), limit of quantification (LOQ). The electrode achieved an outstanding performance of sensitivity (72.06 µAmM^−1^cm^−2^), LOD (174 µM), and LOQ (582 µM) at optimum deposition time. Interference from oxidation of interfering biomolecules such as ascorbic acid, dopamine, and uric acid were negligible compared to glucose. Finally, the fabricated electrode was employed as a pH sensor and displayed a pH sensitivity of 42.26 mV/pH (R = 0.9922). This fabricated ZnO/V_2_O_5_ electrode exhibited high sensitivity and a stable combined temperature, glucose, and pH sensor which is promising for development of multifunctional sensors in next generation wearables.

## 1. Introduction

Recently, wearable devices have been developed and commercialized due to their broad potential and application [1]. They represent the electronic devices that are applied to the human body and skin to monitor how the body is functioning to give the user understanding of themselves [2,3]. Sensor devices represent one of the most influential parts of wearable device technology which has progressively gained attention from both academic and industrial applications [4,5]. Numerous types of sensors including humidity sensors [6], temperature sensors [7], pressure sensors [8], bio-sensors [9], strain sensors [10], pH sensors [11], etc., are researched for wearable application.

Traditionally, electronic devices were fabricated on planar substrates such as glass and metal substrates [12]. These rigid substrate based devices deliver high performances but are not applicable as wearables because of their inflexible nature which is a major drawback [13]. The application of these various sensors to the human body requires flexible substrates with high compatibility to the human body or other surfaces [3]. To overcome the poor flexibility and stretchability of these conventional substrates, flexible substrates such as polyethylene terephthalate (PET) and polydimethylsiloxane (PDMS) have been investigated rather, to substitute rigid and expensive glass and metal substrates [14,15]. Besides planar type substrates, cylindrical fiber type substrates with excellent flexibility have also been developed. Recently, our research group developed a flexible cylindrical fiber shaped temperature sensor based graphene/Ni layer which showed a positive temperature coefficient resistance (TCR) of 0.018 °C^−1^ [16]. Unlike the woven type which has a bundle of fibers, the PET fiber employed in our research group is the monofilament type. The woven type consists of a cluster of fibers fabricated together by weaving, knitting, braiding, etc., which involves high manufacturing costs. Our previously reported works have proved the flexibility and compatibility of the PET fiber as a sensor substrate [17]. In this work, similar flexible PET fiber is employed as a substrate for a multifunctional sensor purpose. A trifunctional sensor that features temperature, glucose, and pH sensing on a PET fiber is designed. Combined precise measurement and self-monitoring of these variables are very critical in the body especially in medicine that necessitates a multifunctional sensor preferably to the individual single sensors [18,19]. They can practically be tested through either direct body contact or using biofluids such as sweat and saliva.

Zinc oxide (ZnO) is the most common oxide material which has continuously been researched due to its several properties suitable for sensor device. ZnO possesses high exciton binding energy (60 meV), high band gap energy (3.37 eV), and a high surface area to volume ratio structure [20]. Liao and coworkers reported ZnO based multifunctional nanosensor which detects strain, temperature, and ultraviolet (UV) on polyurethane (PU) fiber, however, the surface state was very rough [21]. Hasan et al. used direct current (DC) magnetron sputtering for depositing ZnO thin film and applied for temperature sensor [22]. Marie et al. also introduced ZnO nanorods-based glucose sensor on indium tin oxide (ITO) substrate which achieved sensitivity of 10.911 mA/mMcm^2^ and a lower limit of detection of 0.22 µM [23].

Oxides of vanadium exist as VO, VO_2_, V_2_O_3,_ and V_2_O_5_ depending on the oxidation state. Vanadium (V) oxide (V_2_O_5_) is known as an active material for gas sensor, UV detection, catalyst, solar cell, and other electrochromic applications due to its wide optical bandgap, outstanding thermoelectric properties, and good physiochemical thermal stability [24,25,26]. We anticipate a combined ZnO and V_2_O_5_ structure can provide enhanced direct electron transfer through biochemical interfering reactions. Various synthetic methods have been used to prepare ZnO structures (rods, tubes, wires, and sheets) particularly for sensor application which include hydrothermal, solvothermal, microemulsion, and sol gel for sensor application [27]. Notably, hydrothermal is the most beneficial technique as it is simple and mainly focusing on the surface modification with controlled structural morphology. The hydrothermal deposition of V_2_O_5_ on ZnO rods may exhibit good electrical properties due to their large optical band gap, stability, layered morphology, and multivalent behaviors [28]. These properties are crucial for a wide range of medical and industrial applications such as energy storage devices, electrochromic devices, solar cells, photochromic devices, and microbolometers, etc. [29,30].

Binachi group focused on V_2_O_5_ based thin film as a transparent temperature and gas sensor with room temperature chemoresistive properties [31]. Xue et al. reported the piezotronic effect of ZnO based temperature sensors. Recently, Mani et al. also focused on ZnO based microfluidic pH sensors for circulating tumor cells in blood [32]. Based on the above, the ZnO/V_2_O_5_ composite may be suitable for multifunctional sensor applications. Though Rahman et al. reported on a d-glucose sensor based on ZnO/V_2_O_5_ which showed sensitivity of 1.27 × 10^−3^ μAμM^−1^cm^−2^ with 125.25 mM limit of detection. This active material has the potential to be applied to multiple sensor applications [33]. Based on our knowledge, there is no report on ZnO/V_2_O_5_ based multifunctional sensors on a flexible substrate for temperature, glucose and pH combined detections. Upon various ways of seed layer coating, radio frequency (RF) magnetron sputtering was applied in this work for ZnO deposition. A rotating device our research group uniquely designed was utilized. During the deposition process, PET fiber was continuously rotated which led to an even and uniform surface condition. This work presents newly designed ZnO/V_2_O_5_-based sensor coated on cylindrical PET fiber for detecting the variables stated above. This type of multifunctional detectors are important for developing industrial and biomedical applications.

## 2. Materials and Methods

### 2.1. Materials

Analytical grades of zinc nitrate hexahydrate (Zn(NO_3_)_2_·6H_2_O, CAS number: 10196-18-6), hexamethylenetetramine (C_6_H_12_N_4_, CAS number: 100-97-0), ammonium metavanadate (NH_4_VO_3_, CAS number: 7803-55-6), oxalic acid dihydrate (H_2_C_2_O_4_·2H_2_O, CAS number: 6153-56-6), polyvinylidene fluoride (PVDF, CAS number: 24937-79-9), carbon black (CAS number: 1333-86-4), nafion (CAS number: 31175-20-9) and glucose oxidase (GOx, CAS number: 9001-37-0) from *aspergillus niger* were used. Acetone, methanol, sodium hydroxide (NaOH, CAS number: 1310-73-2), hydrochloric acid (HCl, CAS number: 7647-01-0), and phosphate buffered saline (PBS, CAS number: 7647-14-5) were used as solvents. PET fiber (CAS number: 25038-59-9) was also used as substrate. All chemicals, solvents, and substrate were purchased from Sigma Aldrich and used without further purification.

### 2.2. Fabrication of ZnO/V_2_O_5_ on ZnO Sputtered PET Fiber

#### 2.2.1. ZnO Seed Deposition

A bare PET fiber with thickness 300 µm and length 7 cm was employed as a flexible substrate. PET fiber was firstly cut and straightened on a hot plate for 30 min at 100 °C. After forming a straightened PET fiber, it was sequentially sonicated in methanol and acetone for 10 min each. The PET fiber was then finally rinsed with deionized water and dried in an oven at 65 °C for 30 min. The first step was ZnO seed layer coating by RF magnetron sputtering. A rotating device which our research group firstly developed was used during the sputtering procedure. PET fiber was rotated at constant speed of 0.5 rev/s inside the sputter chamber with sputtering conditions; working power of 100W; working pressure of 3.15 × 10^−2^ torr; Ar and O_2_ flow rates of 45 SCCM and 15 SCCM respectively. Various deposition times (10 min, 20 min, and 30 min) were applied to study the effect of seed layer thickness on characteristics of ZnO.

#### 2.2.2. Simultaneous Hydrothermal Growth of ZnO and ZnO/V_2_O_5_

The typical hydrothermal growth of ZnO and ZnO/V_2_O_5_ on the seed layer: 0.1 M equimolar concentration of Zn(NO_3_)_2_.6H_2_O and C_6_H_12_N_4_ were used as precursors. The precursors were mixed together in 70 mL of water and stirred for 24 h. After stirring, the solution was transferred into a Teflon-lined autoclave. The ZnO seed layer on PET fiber was placed in the autoclave and maintained at 95 °C for 8 h. After cooling, part of the obtained samples was washed three times with water and ethanol and finally dried to obtain ZnO microrods. 1.2 g of NH_4_VO_3_ and 0.5 g of H_2_C_2_O_4_.2H_2_O were dissolved in 20 mL of water and stirred for 6 h. The blue color solution obtained was transferred to the above autoclave with part of the solution after first hydrothermal. A PET fiber was placed into the autoclave and kept at the same reaction conditions. After cooling, the resultant solution was washed three times with water and ethanol and finally dried to obtain ZnO/V_2_O_5_ composite.

### 2.3. Material Characterization

The formation of synthesized ZnO and ZnO/V_2_O_5_ particles was confirmed using X-ray diffractometer (Rigaku Ultima IV) with Cu kα radiations (λ = 1.5046 Å) over theta range from 20° to 80°. Further characterizations including field emission scanning electron microscopy (FESEM, JSM-7500F) was used to study the morphology of the prepared powder. FESEM image of PET fiber coated with ZnO was also recorded. The elemental composition was confirmed using energy dispersive X-ray spectroscopy (EDX) and X-ray photoelectron spectroscopy (XPS, Thermo Scientific K-Alpha).

### 2.4. Construction and TCR Measurement of Temperature Sensor on PET Fiber

After the growth of ZnO/V_2_O_5_ composite on PET fiber, the assembled device was placed on a sliding glass. Copper tape was attached to achieve better contact for I-V measurement using Keithley electrometer (Model 6517). The resistance of the composite was measured for five different points (25 °C, 40 °C, 60 °C, 80 °C, and 100 °C) in a voltage range from –5 V to 5 V. Based on the resistance from the I-V curves, TCR was calculated which was utilized as the temperature sensing parameter. To study the reversibility of ZnO and ZnO/V_2_O_5_ temperature sensor, after increasing the temperature to 100 °C, the temperature was maintained for 1 h and the resistance was measured at similar temperature points during cooling.

### 2.5. Construction of Electrochemical Sensor on PET Fiber

All the electrochemical measurements were carried out using Biologic (SP-150) Potentiostat. The ZnO-V_2_O_5_ coated PET fiber was used as a working electrode, Ag/AgCl electrode in 0.2 M KCl as a reference electrode, and a Pt wire electrode as a counter electrode with different concentrations of NaOH and HCl as electrolytes. Prior to the electrochemical test, an electrode was prepared by thoroughly mixing active material (ZnO/V_2_O_5_), carbon black, and PVDF in a mass ratio of 80:10:10. The prepared sample was coated on the ZnO seed layered PET fiber using a dip coater. The fabricated electrode was dried in a vacuum oven at 60 °C and finally used as an electrochemical sensor.

#### 2.5.1. Enzymatic Glucose Sensing Analysis

To fabricate the glucose sensor, glucose oxidase (GOx) was coated on ZnO microrods and ZnO/V_2_O_5_ composite using dip-coating method with a dip-coating speed of 0.5 mm/s. Different concentrations of glucose were mixed with the 17 mM PBS solution solvent employed as the electrolyte. After GOx coating, the array was dried out overnight followed by nafion (5%) coating. Nafion was also coated by dip-coating method with similar dip-coating speed and dried overnight. The prepared electrode was applied as a working electrode for glucose sensing. The current was measured using the electrochemical system with ZnO-V_2_O_5_ coated PET fiber as the working electrode.

#### 2.5.2. pH Sensing Analysis

The ZnO/V_2_O_5_ composite array was also applied in pH sensing for pH levels between 1 and 12 using a potentiometric technique recording the potential difference across the Ag/AgCl reference electrode and ZnO/V_2_O_5_ working electrode. The pH was controlled by using a mixture of NaOH and HCl solvents in different compositions as the electrolyte.

## 3. Results and Discussion

### 3.1. Materials

**Scheme 1:**
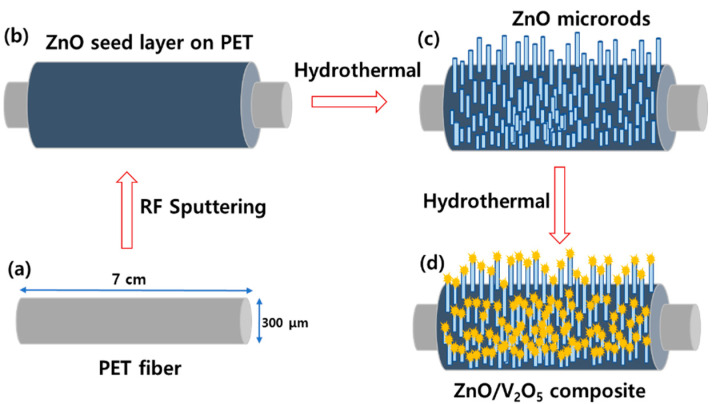
Representation of the fabrication of ZnO microrods and ZnO/V_2_O_5_ composite on a PET fiber. (**a**) bare PET monofilament substrate (**b**) RF sputtered ZnO seeds on PET fiber. Hydrothermal synthesis of (**c**) ZnO microrods and (**d**) ZnO/V_2_O_5_ composite on PET fiber.

Scheme 1 illustrates the schematic representation of the fabrication of ZnO/V_2_O_5_ composite on a single PET fiber. ZnO seed layer was firstly deposited on cleaned cylindrical PET fiber by RF magnetron sputtering at different deposition times (10 min, 20 min, and 30 min) using a rotating device (Figure S1a) designed by our research group in order to ensure a uniform thin film deposition. It is apparent that PET fiber has a cylindrical feature and fine surface condition. (Figures S1b and S2) The rotation speed of the device was maintained at 0.5 rev/s [17,34]. The ZnO/V_2_O_5_ was grown on the ZnO seed layer using a two-step hydrothermal process. A feasible mechanism for the growth of ZnO/V_2_O_5_ microrods is as follows (Equations (1)–(5)):

(1) $$\mathrm{Zn}{\left({\mathrm{NO}}_{3}\right)}_{2}\cdot 6H_{2}O\;\to \;\mathrm{Zn}{\left(\mathrm{OH}\right)}_{2\left(\mathrm{aq}\right)}\;+\;5H_{2}O\;+\;2{\mathrm{NO}}_{3\left(\mathrm{aq}\right)}^{-}$$

(2) $$\mathrm{Zn}{\left(\mathrm{OH}\right)}_{2\left(\mathrm{aq}\right)}\;\to \;{\mathrm{ZnO}}_{\left(s\right)}\;+\;6H_{2}O$$

(3) $$2{\mathrm{NH}}_{4}{\mathrm{VO}}_{3}\;+\;H_{2}C_{2}O_{4}\cdot 6H_{2}O\;\to \;V{\left(\mathrm{OH}\right)}_{4\left(\mathrm{aq}\right)}\;+\;{\left({\mathrm{NH}}_{4}\right)}_{2}C_{2}O_{4\left(s\right)}\;+\;H_{2\left(g\right)}$$

(4) $$2V{\left(\mathrm{OH}\right)}_{4\left(\mathrm{aq}\right)}\;\to \;V_{2}O_{5}\;+\;3H_{2}O\;+\;H_{2\left(g\right)}$$

(5) $$\mathrm{ZnO}\;+\;V_{2}O_{5}\;\to \;\mathrm{ZnO}\cdot V_{2}O_{5}$$

The development of ZnO/V_2_O_5_ composite was accomplished primarily by heterogeneous nucleation of particles in the solution, followed by re-aggregation of the particles to yield rod-like structures according to the Ostwald principle. The microrods re-aggregate with each other and with V_2_O_5_ particles through Van Der Waals interaction and finally form the ZnO/V_2_O_5_ microrods.

**Figure 1:**
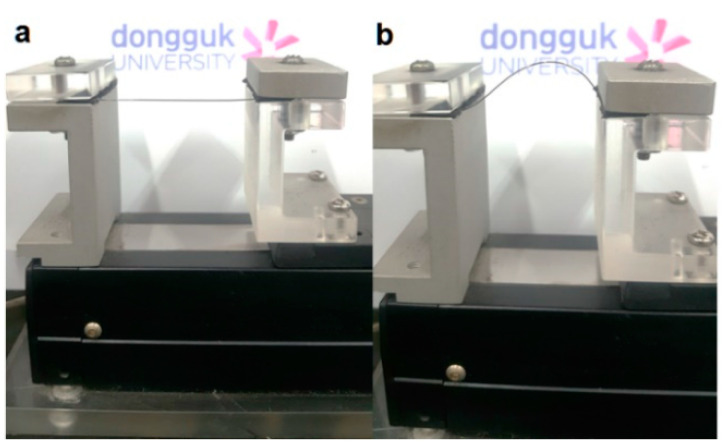
Photographic image of ZnO/V_2_O_5_ deposited PET single fiber (**a**) at straight and (**b**) fullbending conditions.

The flexibility of the coated fiber is confirmed by bending it as shown in Figure 1. A PET fiber of length (L_o_), 7.0 cm was bent at a radius of 18.5 mm. The change in length (∆L) recorded was 2.9 cm and showed an applied strain of 41.4%.

**Figure 2:**
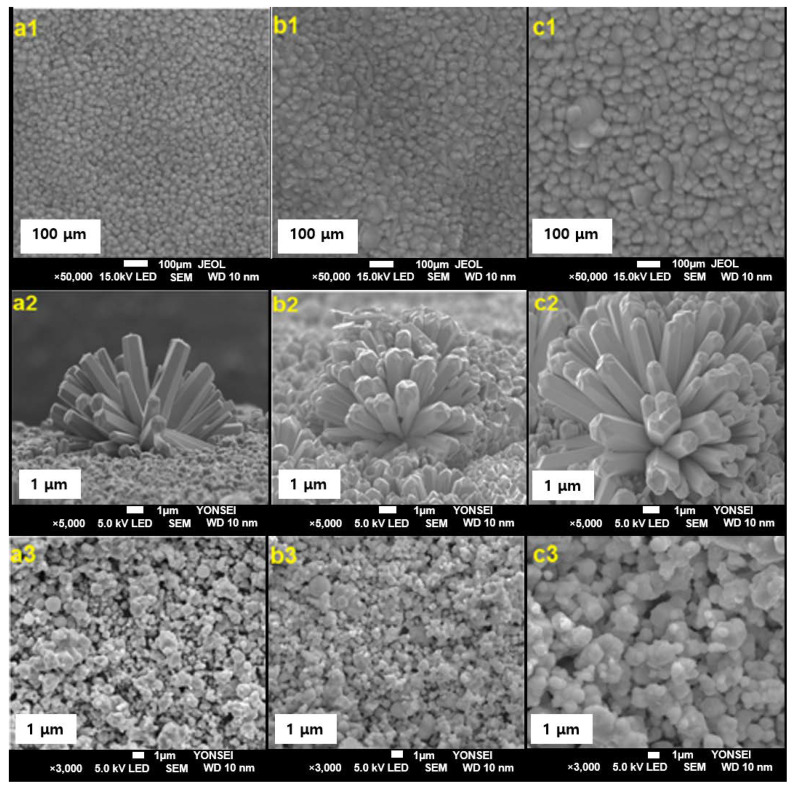
FESEM images of ZnO seed layer (**a1**,**b1**,**c1**); ZnO microrods (**a2**,**b2**,**c2**) and ZnO/V_2_O_5_ composite (**a3**,**b3**,**c3**) for deposition time of 10 min, 20 min, and 30 min respectively.

The surface morphology and the grain size of the samples were observed using FESEM. The FESEM images (Figure 2a1–c1) depict increase in grain size with corresponding reduction in the grain boundary as deposition time increases. The thicknesses of ZnO seeds are approximately 330 μm for 10 min deposition, 360 μm for 20 min deposition and 640 μm for 30 min deposition, respectively [35]. Hydrothermally synthesized ZnO well-oriented growth was also studied from the FESEM characterization (Figure 2a2–c2). It exhibited uniform microrods-like structure and an increased thickness of the rods with increase in deposition time which is due to the grain boundary influences [36]. The actual morphology of ZnO microrods is hexagonal which clearly appears in the image. They are dense and crystalline. The surface grains are composed of regularly shaped grains in a bundle of columnar and triangular shapes. Comparatively, the surface grain size is larger for higher deposition times. Figure 2a3–c3 depicts the surface morphology of ZnO/V_2_O_5_ composite grown on the seed layer. The particles are agglomerated and dense and show different morphology from the pristine ZnO. The microrod morphology of the ZnO/V_2_O_5_ formed influences their intrinsic sensing abilities. The formed microrod structure provides conductive pathways for the electrons, which helps to achieve good electrical characteristics. Figure S3 represents a cross-section of the ZnO/V_2_O_5_ composite on ZnO seeds showing the thickness of the layers. This reveals the rod structure of ZnO/V_2_O_5_ layers. ZnO microrods exhibit a thickness of about 1.02 μm and ZnO/V_2_O_5_ exhibits a layer thickness of 2.31 μm for all the three samples. Represented in Figure S4, coupled EDX spectroscopy was carried out to analyze the elemental composition of the synthesized composite. It depicts the presence of all the expected elements (Zn, V, O) with elemental composition (Zn—28.61%, V—15.04%, and O—56.34%)

**Figure 3:**
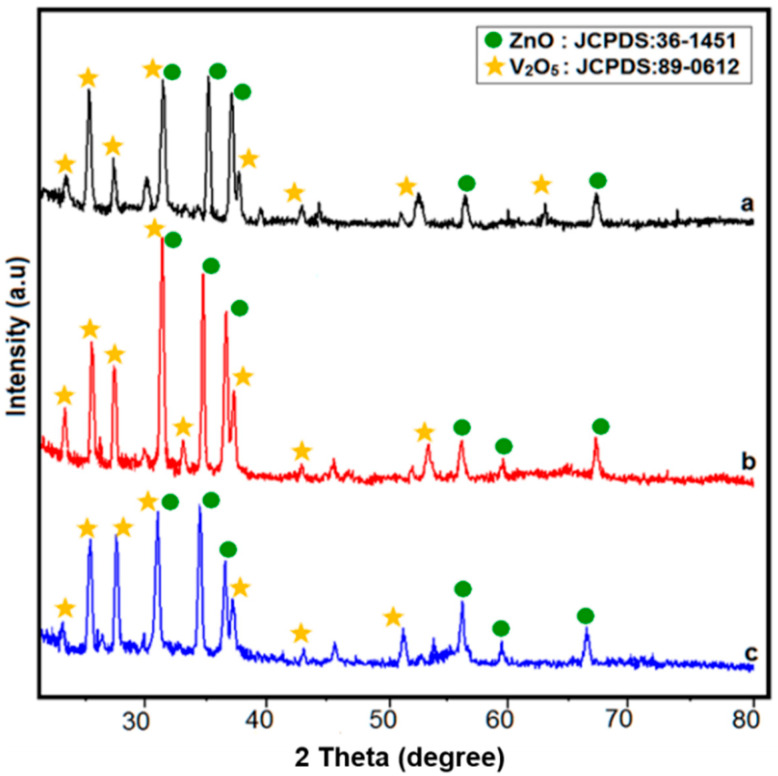
XRD patterns of ZnO/V_2_O_5_ with seed layer deposition time of (**a**) 10 min, (**b**) 20 min, and (**c**) 30 min.

Hydrothermally synthesized samples (ZnO and ZnO/V_2_O_5_) experienced extremely oriented growth which were further studied by XRD and XPS techniques. The XRD studies were plotted in Figure S5 and Figure 3 for ZnO and ZnO/V_2_O_5_, respectively. The expected peaks distinctly appeared which indicate the formation of ZnO microrods for all samples with different deposition times. They showed similar 2θ values of 31.72°, 34.38°, 36.2°, 47.54°, 56.62° and 62.78° independent of the deposition times as represented in Figure S5. Moreover, the crystallinity and orientation of synthesized ZnO microrods were studied by the intensity of ZnO peaks. The peak heights are slightly attenuated for the 10 min ZnO deposition sample signifying lower crystallinity. These peaks indexed the hexagonal phase of the crystalline structure match with the JCPDS card: (36-1451) [28]. No extra peaks confirm a well-crystalline and pure phase ZnO structure. The sharp diffraction peaks in all samples (Figure 3a–c) at the different seed layer sputtering time on PET films authorized the polycrystalline appearance of nanoparticles. The V_2_O_5_ indexed orthorhombic crystalline phase matches with JCPDS: (89-0612) [37]. The other crystalline peaks correspond to the ZnO microrods projecting to increase the crystallinity from sharp peaks. These obtained results indicate that the growth of ZnO microrods are highly oriented to the *z*-axis and vertically aligned. The crystallinity data are consistent with the FESEM characterization results. Also, the intensity of the (002) peak was highest when the deposition time was 20 min. Therefore, ZnO/V_2_O_5_ with 20 min deposition of the seed layer had the best orientation and crystallinity.

**Figure 4:**
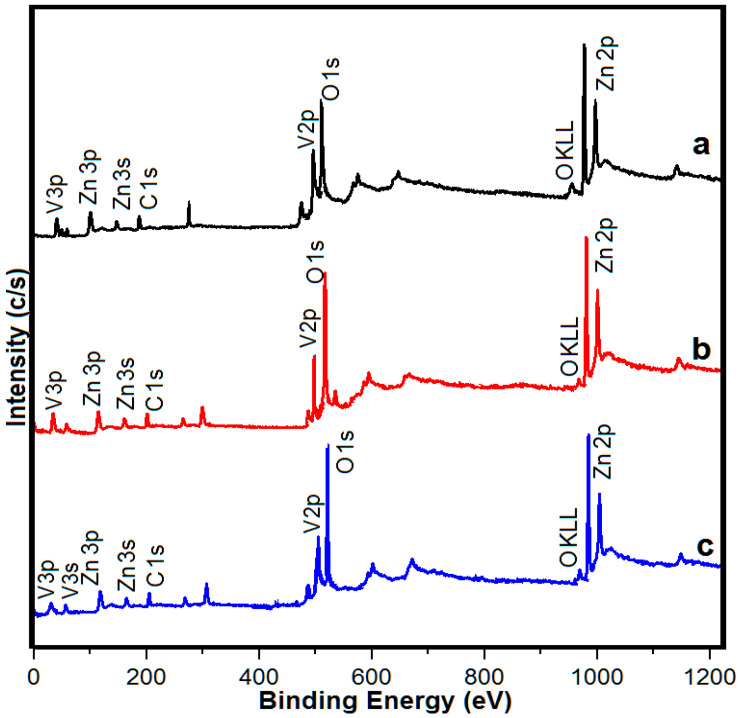
XPS spectra of ZnO/V_2_O_5_ with seed layer deposition time of (**a**) 10 min, (**b**) 20 min, and (**c**) 30 min.

Furthermore, the presence and valence state of the elements in ZnO/V_2_O_5_ were examined with XPS studies (Figure 4). The three overall survey spectra of ZnO/V_2_O_5_ for 10, 20, 30 min deposition times are represented in Figure 4a–c. These XPS graphs indicate typical Zn doublet peaks located around 1022 eV and 1046 eV and correspond to Zn 2p_3/2_ and Zn 2p_1/2_. This binding energy difference may be evidence for the Zn^2+^ oxidation state. The O 1s reveals a prominent peak at 531 eV, which agrees with the Zn surrounded by the nearest oxygen to form a pure ZnO/V_2_O_5_ composite [38]. The typical peaks around 515 eV and 518 eV binding energy were assigned to V 2p_3/2_ and V2p_1/2_ of V_2_O_5_ [39], respectively, which are confirming the successful formation of ZnO/V_2_O_5_ via simultaneous hydrothermal deposition.

### 3.2. Temperature Sensor

**Figure 5:**
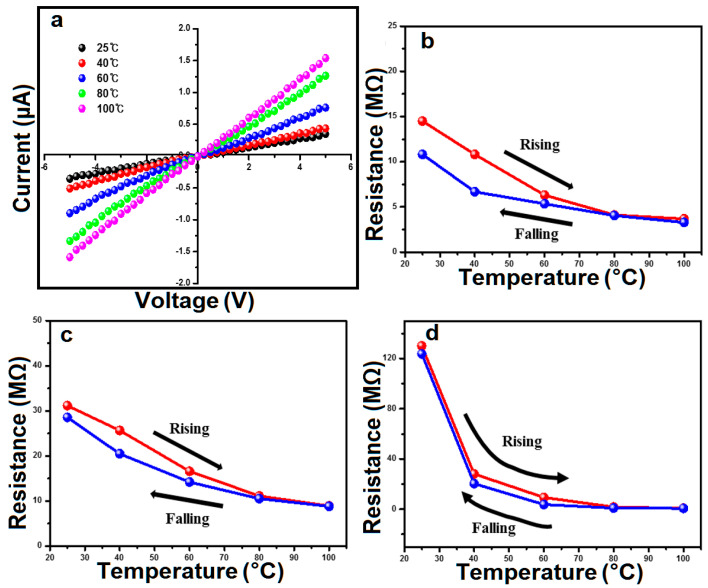
(**a**) Variation of the electrical properties of ZnO/V_2_O_5_ temperature sensor at different temperature conditions. Resistance–temperature relation of ZnO/V_2_O_5_ temperature sensor for seed layer deposition time of (**b**) 10 min, (**c**) 20 min, and (**d**) 30 min.

After the successful synthesis of ZnO/V_2_O_5_ composite on ZnO seed layer, electrical properties were measured for temperature sensing. Figure 5a depicts the current-voltage (I-V) curves within the applied voltage range from −5 V to 5 V at different temperature conditions. The reciprocal of the slope indicates the electrical resistance of the electrode. Resistance values of different temperatures were applied for calculating temperature coefficient of resistance (TCR). TCR defined in Equation (6) indicates the temperature sensitivity of a certain material as a function of resistance,

(6) $$\mathrm{TCR}\;=\;\frac{R_{2}\;-\;R_{1}}{R_{1}\left(T_{2}\;-\;T_{1}\right)}$$

where R_1_ is initial resistance, R_2_ is final resistance, T_1_ is the initial temperature and T_2_ is final temperature. In this work, initial temperature is 25 °C and final temperature is 100° C. Thus, TCR was calculated by measuring the resistance change between 25 °C and 100 °C [16].

The change in resistance corresponding to temperature change is plotted in Figure 5b–d. This observation represents the resistive behavior of the deposited composite on PET fiber. Resistance was measured during the cooling process to understand the reversibility of the temperature sensor. We observed an inverse relationship between resistance and temperature for the cooling similar to the heating process. All three samples showed identical behavior. The final resistance was smaller than the initial resistance, which renders a negative TCR value. This phenomenon is one of the natural behaviors of semiconductor materials. The inverse resistance-temperature relationship is because a rise in thermal energy excites electrons to move from the valence band to conduction band. More electrons jumping leads to higher current and lower resistance [37].

Reversely, the metal heating process may increase the number of vibrating atoms. Random collision of these atoms hinders the passage of electrons which explains the increase in resistance with temperature rise. For 10 min deposition sample, the resistance decreased from 14.49 MΩ to 3.68 MΩ as temperature increased from 25 °C to 100 °C (Figure 5b). In the reversible process, resistance increased from 3.27 MΩ to 10.81 MΩ. Rising TCR is −10.49 × 10^−3^ °C^−1^ and falling TCR is 8.00 × 10^−3^ °C^−1^ respectively. For 20 min deposition sample, resistance decreased from 31.15 MΩ to 8.89 MΩ as temperature increased from 25 °C to 100 °C. Resistance increased from 8.80 MΩ to 28.54 MΩ during the reverse process (Figure 5c). The obtained rising TCR is −10.02 × 10^−3^ °C^−1^ and falling TCR is 8.88 × 10^−3^ °C^−1^. A similar trend was recorded in the third sample; seed layer deposited for 30 min with resistance decreasing from 130.19 MΩ to 0.72 as temperature increased from 25 °C to 100 °C. The condition of temperature decreased from 100 °C to 25 °C and resulted in a resistance rise from 0.61 MΩ to 123.72 MΩ. Rising and falling TCR are −11.09 × 10^−3^ °C^−1^ and 10.82 × 10^−3^ °C^−1^ respectively (Figure 5d). The resistance in the 10 min ZnO seeds sample is a single-valued function of temperature up to 80 °C, above which there is a sudden reduction in sensitivity. A similar trend is observed in the sensor with 20 min deposition time. However, it exhibits a region within a temperature range of 30–40 °C, in which resistance is multivalued at different temperatures, which makes the device unusable for temperature measurements close to the body temperature. Moreover, an increase in deposition time to 30 min displayed a linear resistance–temperature function below 40 °C coupled with high sensitivity which is appropriate for temperature measurements close to the body temperature. In contrast, there is no appreciable change in resistance with temperatures above 60 °C. The average TCR value was highest when the seed layer deposition time was 30 min. However, the linearity of 30 min deposition sample was weaker than other samples. Even though the 30 min seeded sample exhibited a downgraded crystallinity quality compared to the 20 min seeded sample, it showed the best TCR. We speculate that the sensing performance does not depend only on crystallinity but the grain size also maximizes the sensing ability. The 30 min seeded sample exhibited the largest grain size, which provides a larger number of boundaries for temperature sensing. This is reasonable to conclude that the crystallinity, as well the grain size is essential to attain sensing capacities. The crystallinity and grain size of ZnO/V_2_O_5_ composite affected its temperature sensing ability.

### 3.3. Electrochemical Sensor

#### 3.3.1. Glucose Sensor

In the electrode preparation, carbon black and PVDF were mixed with the active material respectively to improve the conductivity and stability upon voltage application. 17 mM PBS employed as a working electrolyte has a pH of 7.4 which lies between the isoelectric points of ZnO of 9.2 and glucose of 4.2 respectively. This renders ZnO surface positively charged and glucose negatively charged. Among the families of enzymes associated with glucose metabolism, GOx is employed in our experiment because of its high activity and availability. In addition, Nafion was coated on the GOx layer to reduce the enzyme leakage. The probable mechanism involves an initial interaction of GOx with glucose. Glucose is converted to gluconolactone and finally to gluconic acid with the release of H_2_O_2_. The H_2_O_2_ decomposes into an oxygen molecule and protons by generating electrons which are responsible for the current-potential characteristic for glucose sensing [33]. The glucose-sensing phenomenon is well elaborated in the following reactions:

(7) $$O_{2}\;\to \;O_{2\;\mathrm{ads}}\left(\mathrm{ZnO}\right)$$

(8) $$O_{2\left(\mathrm{ads}\right)}\left(\mathrm{ZnO}/V_{2}O_{5}\right)\;+\;2e^{-}\left(\mathrm{ZnO}\right)\;\to \;2O_{\left(\mathrm{ads}\right)}^{-}\left(O^{-}/O^{2-}\right)$$

(9) $$\mathrm{Glucose}\;+\;O^{-}\;\overset{\mathrm{GOx}}{\to }\;\mathrm{Glucono}\;-\;\delta \;-\;\mathrm{lactone}\;+\;2e^{-}$$

Initially, the accessible O in the glucose atmosphere was adsorbed onto the surface of ZnO/V_2_O_5_ as an oxygen (O) monolayer. The neutral O was then activated to (O^−^/O^2−^). Finally, glucono-δ-lactone is produced from glucose oxidation through a biochemical process catalyzed by GOx. The sensing electrode detects the electron transfer by reading the current. A schematic diagram of the glucose sensor is represented in Scheme S2 in the supplementary materials. The high surface area of ZnO/V_2_O_5_ provides a good aspect ratio for GOx immobilization and contributes to the high current detection by influencing the electron transfer through structural arrangement [40].

**Figure 6:**
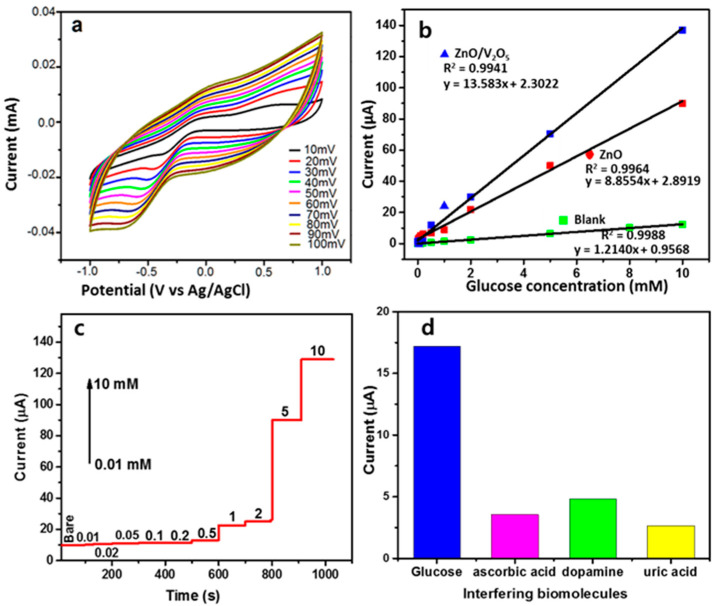
(**a**) Cyclic voltammetry curve of ZnO/V_2_O_5_ electrode at different scan rate applied in glucose sensing, (**b**) calibration plot of blank, ZnO and ZnO/V_2_O_5_ glucose sensors at +0.8 V with a straight line representing the linear fit for ZnO seed layer deposited on PET for 20 min, (**c**) current-time response monitoring according to increasing glucose concentration towards ZnO/V_2_O_5_ electrode, and (**d**) bar diagram representation of the interference effect at +1.0 V.

In Figure S6, the CV curves were first plotted for GOx (without V_2_O_5_ and ZnO/V_2_O_5_) within a large potential window (0–1.5 V vs. Ag/AgCl). An observation of a hump clearly shows that H_2_O_2_ oxidation is around 600 mV [41]. The CV curves were also measured within an applied potential from −1.0 V to 1.0 V with 0.1 mM with glucose as the analyte in 17 mM PBS electrolyte in the presence of ZnO and ZnO/V_2_O_5_ electrodes. The CV curves illustrated in Figure 6a show characteristic cathodic and anodic peaks. Increasing the scan rate resulted in a positive potential shift of the cathodic peak and a negative potential shift of the anodic peak [30]. Interestingly, two cathodic peaks were observed with one considerably below −100 mV and the other slightly above 600 mV. However, a calibrated potential higher than the latter was selected to construct the calibration curve because detection of enzymatic reaction (oxidation of H_2_O_2_) takes place at 600 mV at Pt electrode [33].

To study glucose sensitivity, a calibration curve (both with and without electrodes) was plotted at +0.8 V as shown in Figure 6b for glucose concentration (100 µM–10 mM) which was observed to exhibit a linear characteristic. ZnO layer deposition was selected with 10 min and 30 min represented in Figure S7. After linear fitting, correlation coefficient (R^2^) is 0.9964 for ZnO microrods and 0.9941 for composite. The right current was calculated as the recorded current minus the blank signal. Glucose sensitivity, limit of detection (LOD), and limit of quantification (LOQ) were calculated from the equations;

(10) $$\mathrm{Sensitivity}\;=\;\frac{m}{A}$$

(11) $$\mathrm{LOD}\;=\;\frac{\left(3\;\times \;\mathrm{SD}\right)}{m}$$

(12) $$\mathrm{LOQ}\;=\;\frac{\left(10\;\times \;\mathrm{SD}\right)}{m}$$

where, A—active surface area of PET working electrode, m—slope of the calibration curve, and SD—standard deviation at the calibrated potential (0.79).

The active surface area of PET fiber was calculated; A = 2πRL, where R stands for radius and L stands for the active length of PET fiber, A = 2 × π × 0.015 cm × 2 cm = 0.1885 cm^2^. Table S1 shows a summary of the sensitivity, limit of detection (LOD), and limit of quantification (LOQ) of both ZnO and ZnO/V_2_O_5_ electrodes at different deposition times. We observe that the seed layer deposited for 20 min demonstrates optimum glucose sensing with highest sensitivity and lowest LOD. High sensitivity (72.06 μAmM^−1^cm^−2^), LOD (0.174 mM), and LOQ (0.582 mM) were recorded for the proposed ZnO/V_2_O_5_ based glucose sensor on 20 min seed layer PET fiber. ZnO on the other hand exhibited lower performance of sensitivity (46.98 μAmM^−1^cm^−2^), LOD (0.268 mM), and LOQ (0.892 mM) at equivalent measuring conditions all in a linear dynamic range (LDR) of 10 μM–10 mM. Figure 6c shows a continuous increase in current with increase in glucose concentration. The glucose sensing performance of ZnO/V_2_O_5_ is compared with several previously published works as represented in Table 1. It is noticeable that the I-V design is reliable and offers remarkable glucose sensitivity with low LOD. This renders the proposed electrode a potential glucose sensor that can be integrated in next generation sensors.

To study mechanical stability and strength of ZnO/V_2_O_5_, the PET fiber array was bent repetitively. The curvature of bend was 0.048 mm^−1^ with an applied strain of bending 36.7%. After bending, the current was measured at different glucose concentrations and the glucose sensitivity analysis at calibrated potential, +1.0 V was represented in Figure S9a–c. The ZnO/V_2_O_5_ based electrochemical glucose sensor exhibited 12.2430 μAmM^−1^cm^−2^ (R^2^ = 0.9628), 11.2546 μAmM^−1^cm^−2^ (R^2^ = 0.9367) and (5.9576 μAmM^−1^cm^−2^ R^2^ = 0.9533) after 50 cycles, 100 cycles and 200 cycles of bending. This represents 16.99%, 15.62%, and 8.27% of the initial sensitivity. Figure S9d depicts a summary of mechanical stability of the glucose sensor. The sensitivity loss may be due to bending of fiber substrate which resulted in cracks on ZnO/V_2_O_5_ electrode rendering the linearity of glucose sensitivity and conductivity is aggravated [44].

Interference examination is one of the most important tools in analytical science to differentiate biomolecules showing similar physiological characteristics. Dopamine, ascorbic, and uric acid were employed as interfering constituents towards ZnO/V_2_O_5_ glucose sensors in this study [45]. The I-V characteristic was recorded upon addition of (1 mL, 5 mM) each of dopamine, ascorbic acid, and uric acid in (15 mL, 17 mM) PBS solution. Table 2 shows a summary of the current response towards the addition of (0.5 mL, 100 µM) each of the interfering molecules into (10 mL, 17 mM) of PBS solution. The interference effects of these biomolecules using glucose as reference are calculated at calibrated potential (+1.0 V) and summarized in Figure 6d. It is observed dopamine, ascorbic and uric acid showed minimal interference towards ZnO/V_2_O_5_ which renders the proposed sensor selective to glucose detection with high sensitivity.

#### 3.3.2. pH Sensor

**Figure 7:**
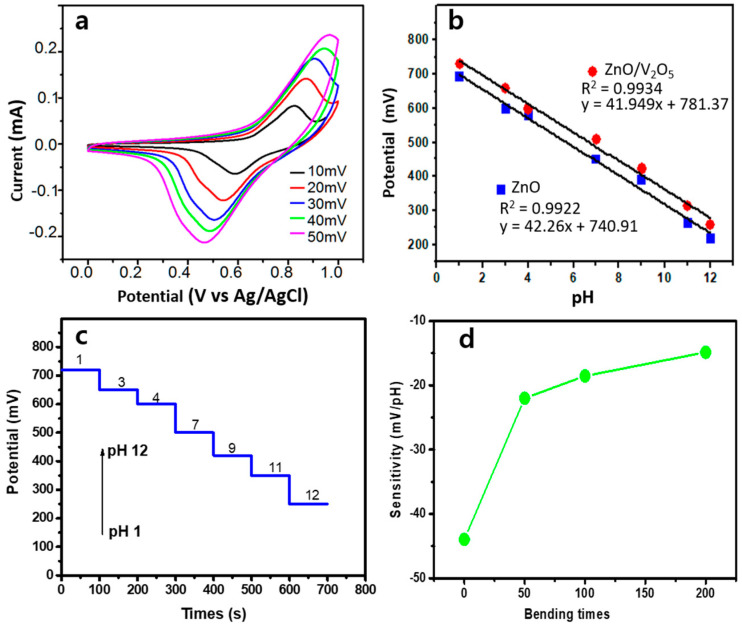
(**a**) Cyclic voltammetry curve of ZnO/V_2_O_5_ electrode at different scan rates in NaOH/HCl mixture of pH 4, (**b**) calibration plot for ZnO and ZnO/V_2_O_5_ pH sensors with a straight line representing the linear fit, (**c**) potential–time response obtained on increasing the pH of NaOH/HCl electrolyte at ZnO/V_2_O_5_ electrode, and (**d**) calculated sensitivity after various bending cycles to validate the mechanical stability of ZnO/V_2_O_5_ pH sensors.

In addition to the glucose sensing property, ZnO/V_2_O_5_ showed sensitivity toward pH. As previously mentioned, large surface area-to-volume ratio, makes ZnO an excellent material for pH sensing. Rather than 17 mM PBS, NaOH and HCl solutions containing different concentrations were used for the electrolyte. Cyclic voltammetry curves at different scan rates of the prepared electrode in pH 4 solution are depicted in Figure 7a. High anodic peak and cathodic peak currents are apparent which are an indication of a promising pH sensing characteristic. This type of metal oxide is a typical amphoteric oxide material that reacts with both acidic solution and basic solution. [46] Zn shows electropositive features in acidic medium and electronegative features in alkaline medium. Figure 7b explains the potential variation with changes in pH. Performing a linear fit produced the slope which is an indication of the pH sensitivity. A high sensitivity of 41.95 mV/pH (R^2^ = 0.9934) was recorded for ZnO/V_2_O_5_ composite and 42.26 mV/pH of sensitivity (R^2^ = 0.9922) for ZnO which represent 71.1% and 71.6% of the ideal Nernstian limit (59.14 mV/pH). From the Nernst equation, the number of electrons transferred during the redox reaction of ZnO and ZnO/V_2_O_5_ is responsible for the lower response compared to only one electron for monovalent ions (H^+^). The potential–time response in different pH solutions towards ZnO/V_2_O_5_ and ZnO are indicated in Figure 7c and Figure S10a respectively. It can be deduced that ZnO and ZnO/V_2_O_5_ composite showed a high potential in an acidic environment and low potential in a basic environment.

Potential change upon repetitive bending was plotted in Figure S10b,d for different bending cycles. Potential value and pH sensitivity slightly decreased after repetitive bending test. The curvature of bend was maintained at 0.048 mm^−1^ with an applied strain of bending 36.7% same as in the bending test on the glucose sensor. The exhibited pH sensitivity is 21.99 mV/pH (R^2^ = 0.8692), 18.54 mV/pH (R^2^ = 0.9481) and 14.85 mV/pH (R^2^ is 0.9143) after 50 cycles of bending, 100 cycles bending and 200 cycles of bending respectively. As shown in Figure 7d, there is a drastic reduction in the sensitivity after 50 cycles of bending with slight reduction for subsequent bends. This observation, we attribute to a sudden loss of stability due to the stress induced in the PET fiber. Overall performance of the ZnO microrods coated PET fiber exhibits reasonable pH sensitivity performances of 25% of the Nernst limit after 200 cycles bending conditions.

## 4. Conclusions

This work explains fabrication of a highly flexible trifunctional sensor which features temperature, glucose, and pH detection on a cylindrical PET fiber. Uniform and even surface deposition of ZnO layer was obtained by using a rotating device inside the sputter chamber. The main component of the sensitive layer, ZnO/V_2_O_5_ was synthesized using hydrothermal reaction and deposited on the ZnO seed layer. The pristine ZnO/V_2_O_5_ hexagonal-like rods were characterized using XRD, XPS, FESEM, and CV. Compared to single ZnO microrods, the ZnO/V_2_O_5_ electrode exhibits better electrical activity towards temperature and electrochemical sensors. It recorded better reversibility for both rising and falling conditions with maximum TCR of 0.0111 °C^−1^ at 30 min ZnO layer deposition. An outstanding glucose sensing performance was achieved with ZnO/V_2_O_5_. It displayed a linear dependence (R = 0.9941) in a glucose concentration between (10 µM–10 mM) with a sensitivity of 72.06 µAmM^−1^cm^−2^ and a limit of detection at 174 µM at optimum 20 min deposition time. ZnO/V_2_O_5_ glucose sensor could retain only 8.3% of sensing ability after 200 bending cycles. Ascorbic acid, dopamine, and uric acid displayed negligible interference making ZnO/V_2_O_5_ selective to glucose molecules. Also, the fabricated electrode exhibited pH sensitivity of 42.26 mV/pH (R^2^ = 0.9922) which is about 72% of the Nernstian response. After repetitive bending, the device showed good mechanical stability which appeals to its potential to be developed as a multifunctional (temperature, glucose, and pH) sensor. The sensor would be installed outside the human body. It could be woven into comfortable, form-fitting, or stretchy fabrics to remotely track temperature, glucose, and pH signals.

## Figures and Tables

**Table 1:** Comparison of glucose sensing ability of ZnO/V_2_O_5_ electrode with previously published for sensor application.

Electrode	Method of Detection	LOD ^1^ (µM)	LDR ^2^ (µM)	Sensitivity (μAmM^−1^cm^−2^)	Reference
MnO_2_/MWCNT	Amperometry	-	10–28,000	33.19	[42]
V_2_O_5_ nanoenzymes	Chronoamperometry	10	10–2000	-	[43]
ZnO NR	Amperometry	0.22	-	0.0109	[23]
ZnO/V_2_O_5_ NR	I-V	125,250	1–1000	1.27	[33]
ZnO/V_2_O_5_ on PET	Amperometry	174	10–10,000	72.06	This work

^1^ Limit of Detection. ^2^ Linear Dynamic Range.

**Table 2:** Interference effect of similar biomolecules towards ZnO/V_2_O_5_ sensing electrode.

IBM	CR, uA				IE (%)
	R1	R2	R3	Avg	
G	20.32	18.68	12.64	17.21	100
AA	3.20	4.31	3.11	3.54	20.6
D	5.61	4.64	4.16	4.80	28.9
UA	1.93	3.12	2.86	2.64	15.3

(0.5 mL, 100 µM) IBM—interfering molecules, IE—interfering effect, CR—current recorded, G–glucose, AA—ascorbic acid, D—dopamine, UA—uric acid, R—reading, Avg—average.

## Data Availability

The data presented in this work are available on request from the corresponding author.

## Supplementary Materials

The following are available online at https://www.mdpi.com/article/10.3390/s21072559/s1, Figure S1: photographic image of (a) sputtering jig for rotating PET fiber (b) PET fiber and ZnO/V_2_O_5_ coated PET fiber, Figure S2: SEM image of PET fiber, Figure S3: cross-sectional SEM image of ZnO/V_2_O_5_ composite on ZnO seeds. Figure S4: SEM-EDX pattern of ZnO/V_2_O_5_ showing the elemental composition. Figure S5: XRD patterns of ZnO microrods deposited with seed layer deposition time of (a) 10 min (b) 20 min, and (c) 30 min, Scheme S2: schematic representation of the electrochemical sensor testing. RE, CE, and WE represent reference electrode, counter electrode, and working electrode respectively, Figure S6: CV profiles for GOx in the absence of ZnO or ZnO/V_2_O_5_ (blank solution) at different scan rates, Figure S7: calibration plot of ZnO and ZnO/V_2_O_5_ glucose sensors at +0.8 V with a straight line representing the linear fit for (a) 10 min and (b) 30 min ZnO seed layer deposition times on PET substrates, Figure S8: current–time response monitoring according to increasing glucose concentration towards ZnO electrode, Figure S9: calibration plot for ZnO/V_2_O_5_ glucose sensing at +1.0 V with a straight line representing the linear fit for ZnO seed layer deposited on PET for 20min after (a) 50 cycles (b) 100 cycles (c) 200 cycles (d) variation of calculated sensitivity after various bending cycles, Figure S10: (a) potential–time response obtained on increasing the pH of NaOH/HCl electrolyte for ZnO electrode. Calibration plot for ZnO/V_2_O_5_ pH sensors with a straight line representing the linear fit after (a) 50 cycles (b) 100 cycles (c) 200 cycles of repetitive bending, Table S1 sensitivity, limit of detection (LOD), and limit of quantification (LOQ) of ZnO and ZnO/V_2_O_5_ glucose sensor on ZnO seed layer at different deposition times.

## References

[1] Khan Y., Thielens A., Muin S., Ting J., Baumbauer C., Arias A.C. (2020). A New Frontier of Printed Electronics: Flexible Hybrid Electronics. Adv. Mater..

[2] Poh M.Z., Swenson N.C., Picard R.W. (2010). A wearable sensor for unobtrusive, long-term assessment of electrodermal activity. IEEE Trans. Biomed. Eng..

[3] Gao W., Emaminejad S., Nyein H.Y.Y., Challa S., Chen K., Peck A., Fahad H.M., Ota H., Shiraki H., Kiriya D. (2016). Fully integrated wearable sensor arrays for multiplexed in situ perspiration analysis. Nature.

[4] Li Y.Q., Zhu W.B., Yu X.G., Huang P., Fu S.Y., Hu N., Liao K. (2016). Multifunctional Wearable Device Based on Flexible and Conductive Carbon Sponge/Polydimethylsiloxane Composite. ACS Appl. Mater. Interfaces.

[5] Ryu S., Lee P., Chou J.B., Xu R., Zhao R., Hart A.J., Kim S.G. (2015). Extremely Elastic Wearable Carbon Nanotube Fiber Strain Sensor for Monitoring of Human Motion. ACS Nano.

[6] Wang Y., Zhang L., Zhang Z., Sun P., Chen H. (2020). High-Sensitivity Wearable and Flexible Humidity Sensor Based on Graphene Oxide/Non-Woven Fabric for Respiration Monitoring. Langmuir.

[7] Yu Y., Peng S., Blanloeuil P., Wu S., Wang C.H. (2020). Wearable Temperature Sensors with Enhanced Sensitivity by Engineering Microcrack Morphology in PEDOT:PSS-PDMS Sensors. ACS Appl. Mater. Interfaces.

[8] He J., Zhang Y., Zhou R., Meng L., Chen T., Mai W., Pan C. (2020). Recent advances of wearable and flexible piezoresistivity pressure sensor devices and its future prospects. J. Mater..

[9] Parlak O., Curto V.F., Ojeda E., Basabe-Desmonts L., Benito-Lopez F., Salleo A. (2019). Wearable biosensors and sample handling strategies. Wearable Bioelectronics.

[10] Tan C., Dong Z., Li Y., Zhao H., Huang X., Zhou Z., Jiang J.W., Long Y.Z., Jiang P., Zhang T.Y. (2020). A high performance wearable strain sensor with advanced thermal management for motion monitoring. Nat. Commun..

[11] Manjakkal L., Dervin S., Dahiya R. (2020). Flexible potentiometric pH sensors for wearable systems. RSC Adv..

[12] Huang Q., Zhu Y. (2019). Printing Conductive Nanomaterials for Flexible and Stretchable Electronics: A Review of Materials, Processes, and Applications. Adv. Mater. Technol..

[13] Yuen J.D., Baingane A., Hasan Q., Shriver-Lake L.C., Walper S.A., Zabetakis D., Breger J.C., Stenger D.A., Slaughter G. (2019). A Fully-Flexible Solution-Processed Autonomous Glucose Indicator. Sci. Rep..

[14] Xu J., Su W., Li Z., Liu W., Liu S., Ding X. (2017). A modularized and flexible sensor based on MWCNT/PDMS composite film for on-site electrochemical analysis. J. Electroanal. Chem..

[15] Bi C., Chen B., Wei H., DeLuca S., Huang J. (2017). Efficient Flexible Solar Cell based on Composition-Tailored Hybrid Perovskite. Adv. Mater..

[16] Hilal A.M., Han B.J.I. (2020). Development of a Highly Flexible and Durable Fiber-Shaped Temperature Sensor based on Graphene/Ni Double-Decked Layer for Wearable Devices. IEEE Sens. J..

[17] Eom T.H., Han J.I. (2017). The effect of the nickel and chromium concentration ratio on the temperature coefficient of the resistance of a Ni–Cr thin film-based temperature sensor. Sens. Actuators A Phys..

[18] Kaidarova A., Khan M.A., Marengo M., Swanepoel L., Przybysz A., Muller C., Fahlman A., Buttner U., Geraldi N.R., Wilson R.P. (2019). Wearable multifunctional printed graphene sensors. npj Flex. Electron..

[19] Yao S., Zhu Y. (2014). Wearable multifunctional sensors using printed stretchable conductors made of silver nanowires. Nanoscale.

[20] Girija K.G., Somasundaram K., Topkar A., Vatsa R.K. (2016). Highly selective H_2_S gas sensor based on Cu-doped ZnO nanocrystalline films deposited by RF magnetron sputtering of powder target. J. Alloys Compd..

[21] Liao X., Liao Q., Zhang Z., Yan X., Liang Q., Wang Q., Li M., Zhang Y. (2016). A Highly Stretchable ZnO@Fiber-Based Multifunctional Nanosensor for Strain/Temperature/UV Detection. Adv. Funct. Mater..

[22] Hasan S.A., Gibson D., Song S., Wu Q., Ng W.P., McHale G., Dean J., Fu Y.Q. ZnO thin film based flexible temperature sensor. Proceedings of the IEEE Sensors.

[23] Marie M., Mandal S., Manasreh O. (2015). An Electrochemical Glucose Sensor Based on Zinc Oxide Nanorods. Sensors.

[24] Ahmad Bhat B., Khan G.R., Asokan K. (2015). Role of substrate effects on the morphological, structural, electrical and thermoelectrical properties of V2O5 thin films. RSC Adv..

[25] Pan G.X., Xia X.H., Cao F., Chen J., Zhang Y.J. (2014). Carbon cloth supported vanadium pentaoxide nanoflake arrays as high-performance cathodes for lithium ion batteries. Electrochim. Acta.

[26] Liu H., Gao Y., Zhou J., Liu X., Chen Z., Cao C., Luo H., Kanehira M. (2014). Growth of oriented vanadium pentaoxide nanostructures on transparent conducting substrates and their applications in photocatalysis. J. Solid State Chem..

[27] Kolodziejczak-Radzimska A., Jesionowski T. (2014). Zinc oxide-from synthesis to application: A review. Materials.

[28] Kim H., Pak Y., Jeong Y., Kim W., Kim J., Jung G.Y. (2018). Amorphous Pd-assisted H_2_ detection of ZnO nanorod gas sensor with enhanced sensitivity and stability. Sens. Actuators B Chem..

[29] Jin W., Yan S., An L., Chen W., Yang S., Zhao C., Dai Y. (2015). Enhancement of ethanol gas sensing response based on ordered V2O5 nanowire microyarns. Sens. Actuators B Chem..

[30] Vijayakumar Y., Mani G.K., Ponnusamy D., Shankar P., Kulandaisamy A.J., Tsuchiya K., Rayappan J.B.B., Ramana Reddy M.V. (2018). V2O5 nanofibers: Potential contestant for high performance xylene sensor. J. Alloys Compd..

[31] Kesim Y.E., Battal E., Tanrikulu M.Y., Okyay A.K. (2014). An all-ZnO microbolometer for infrared imaging. Infrared Phys. Technol..

[32] Xue F., Zhang L., Tang W., Zhang C., Du W., Wang Z.L. (2014). Piezotronic effect on ZnO nanowire film based temperature sensor. ACS Appl. Mater. Interfaces.

[33] Rahman M.M., Hussain M.M., Asiri A.M. (2019). D-Glucose sensor based on ZnO/V_2_O_5_ NRs by an enzyme-free electrochemical approach. RSC Adv..

[34] Eom T.H., Han J.I. (2017). Resistive behavior of Ni thin film on a cylindrical PET monofilament with temperature for wearable computing devices. Sens. Actuators A Phys..

[35] Anusha J.R., Kim H.J., Fleming A.T., Das S.J., Yu K.H., Kim B.C., Raj C.J. (2014). Simple fabrication of ZnO/Pt/chitosan electrode for enzymatic glucose biosensor. Sens. Actuators B Chem..

[36] Zhang Q.G., Zhang X., Cao B.Y., Fujii M., Takahashi K., Ikuta T. (2006). Influence of grain boundary scattering on the electrical properties of platinum nanofilms. Appl. Phys. Lett..

[37] Wang Y.T., Whang W.T., Chen C.H. (2015). Hollow V_2_O_5_ nanoassemblies for high-performance room-temperature hydrogen sensors. ACS Appl. Mater. Interfaces.

[38] Ahmad R., Tripathy N., Khan M.Y., Bhat K.S., Ahn M.S., Hahn Y.B. (2016). Ammonium ion detection in solution using vertically grown ZnO nanorod based field-effect transistor. RSC Adv..

[39] Wu Y., Gao G., Yang H., Bi W., Liang X., Zhang Y., Zhang G., Wu G. (2015). Controlled synthesis of V_2_O_5_/MWCNT core/shell hybrid aerogels through a mixed growth and self-assembly methodology for supercapacitors with high capacitance and ultralong cycle life. J. Mater. Chem. A.

[40] Fang B., Zhang C., Wang G., Wang M., Ji Y. (2011). A glucose oxidase immobilization platform for glucose biosensor using ZnO hollow nanospheres. Sens. Actuators B Chem..

[41] Heller A., Feldman B. (2008). Electrochemical glucose sensors and their applications in diabetes management. Chem. Rev..

[42] Chen J., Zhang W.D., Ye J.S. (2008). Nonenzymatic electrochemical glucose sensor based on MnO_2_/MWNTs nanocomposite. Electrochem. Commun..

[43] Sun J., Li C., Qi Y., Guo S., Liang X. (2016). Optimizing colorimetric assay based on V_2_O_5_ nanozymes for sensitive detection of H_2_O_2_ and glucose. Sensors.

[44] Zhang Y., Pan T., Yang Z. (2020). Flexible polyethylene terephthalate/polyaniline composite paper with bending durability and effective electromagnetic shielding performance. Chem. Eng. J..

[45] Hussain M.M., Asiri A.M., Arshad M.N., Rahman M.M. (2018). Fabrication of a Ga^3+^ sensor probe based on methoxybenzylidenebenzenesulfonohydrazide (MBBSH) by an electrochemical approach. New J. Chem..

[46] Mani G.K., Morohoshi M., Yasoda Y., Yokoyama S., Kimura H., Tsuchiya K. (2017). ZnO-Based Microfluidic pH Sensor: A Versatile Approach for Quick Recognition of Circulating Tumor Cells in Blood. ACS Appl. Mater. Interfaces.

