# Supplementary material for: Potentiometric Performance of a Highly Flexible-Shaped Trifunctional Sensor Based on ZnO/V2O5 Microrods
Source: Sensors (Basel). 2021 Apr 6;21(7):2559. doi: 10.3390/s21072559 (PMC8038666; doi:10.3390/s21072559)
Supplement: Supplementary file 1 [file sensors-21-02559-s001.pdf]

Potentiometric Performance of a Highly Flexible Fiber-Shaped Trifunctional Sensor Based on ZnO/V_2O_5 Microrods

Alfred Bekoe Appiagyei, Jeong In Han*

Department of Chemical and Biochemical Engineering, Dongguk University, 04620, Seoul, South Korea

*Corresponding author: TEL: +82-2-2260-3364, Email: hanji@dongguk.edu

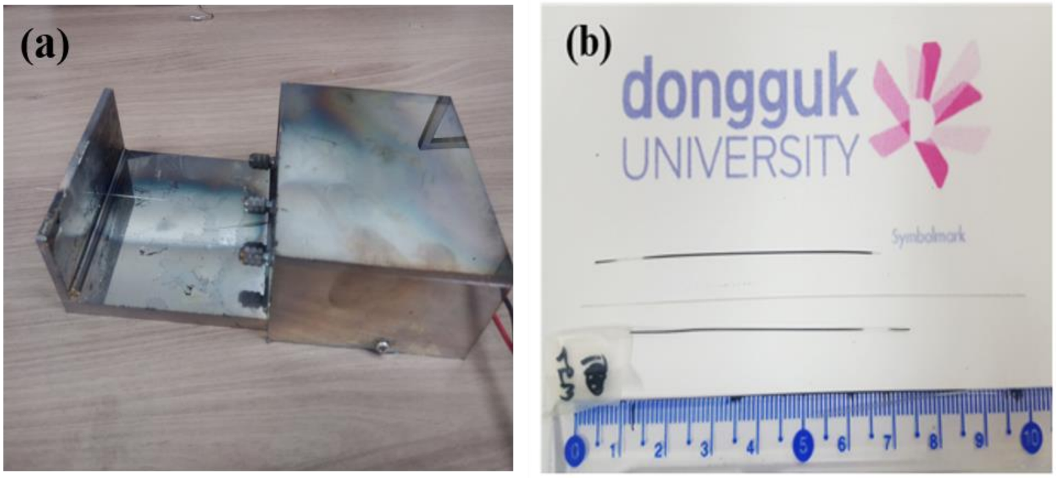

Figure S1: Photographical image of (a) Sputtering jig for rotating PET fibre (b) PET fiber and ZnO/V_2O_5 coated PET fiber

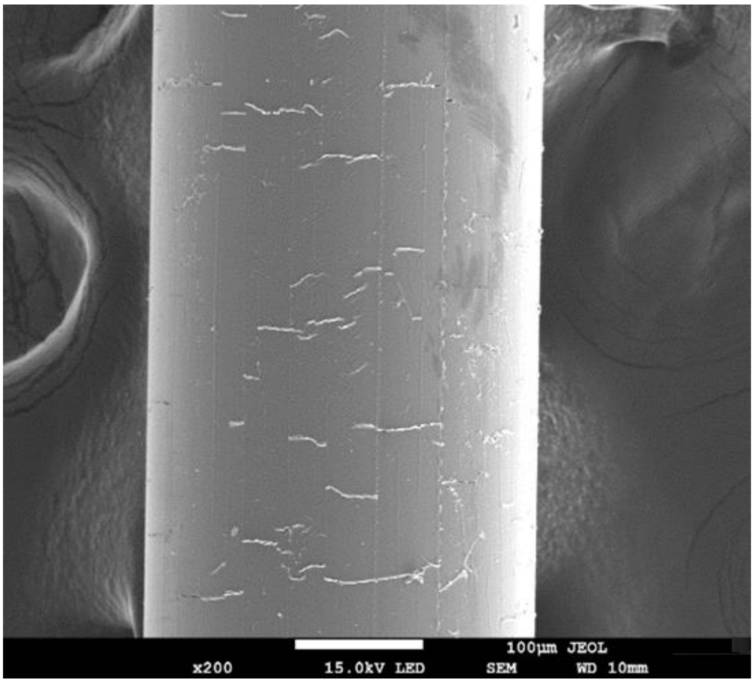

Figure S2 SEM image of PET fiber

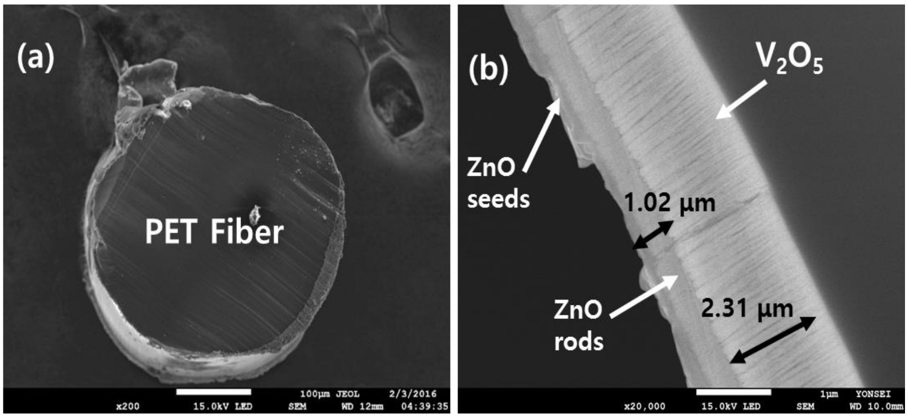

Figure S3: Cross-sectional SEM image of ZnO/V_2O_5 composite on ZnO seeds sputtered on PET fiber.

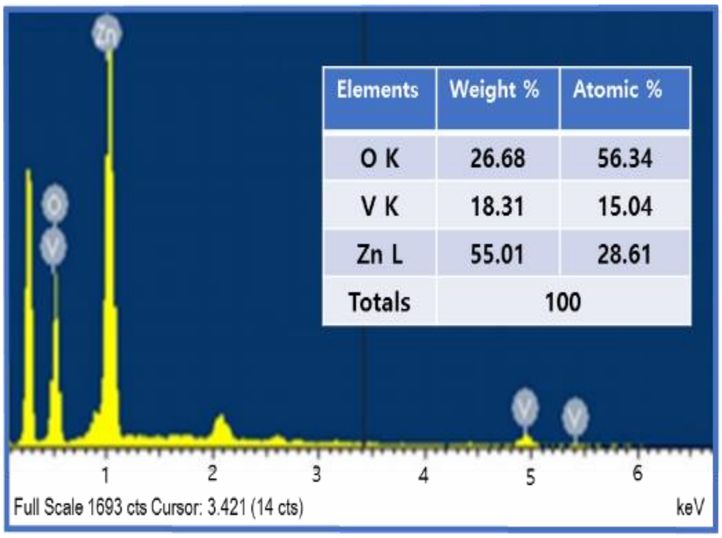

Figure **S4:** SEM-EDX pattern of ZnO/V_2O_5 showing the elemental composition.

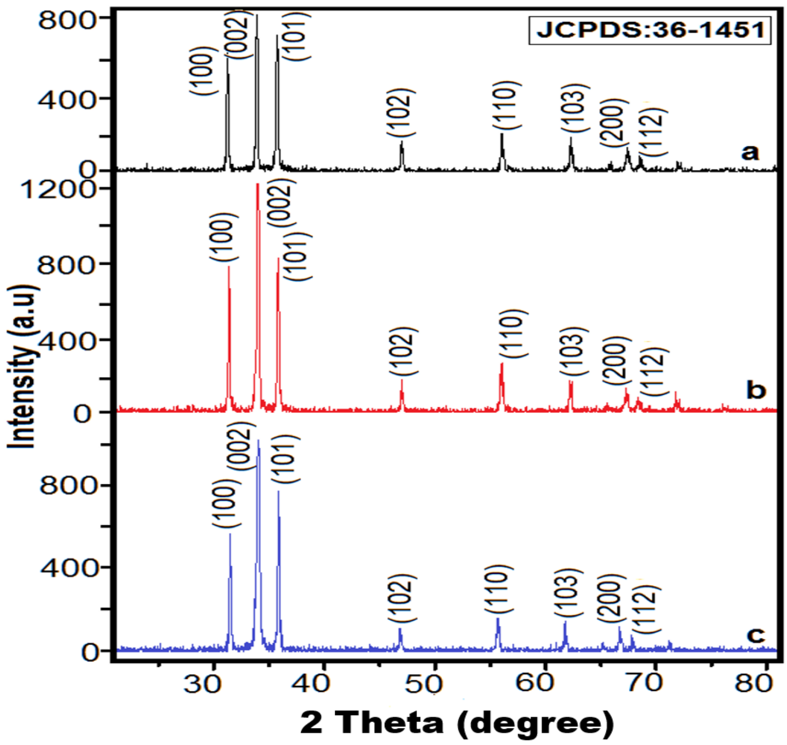

Figure S5. XRD patterns of ZnO microrods deposited with seed layer deposition time of (a) 10 min (b) and 20 min (c) 30 min.

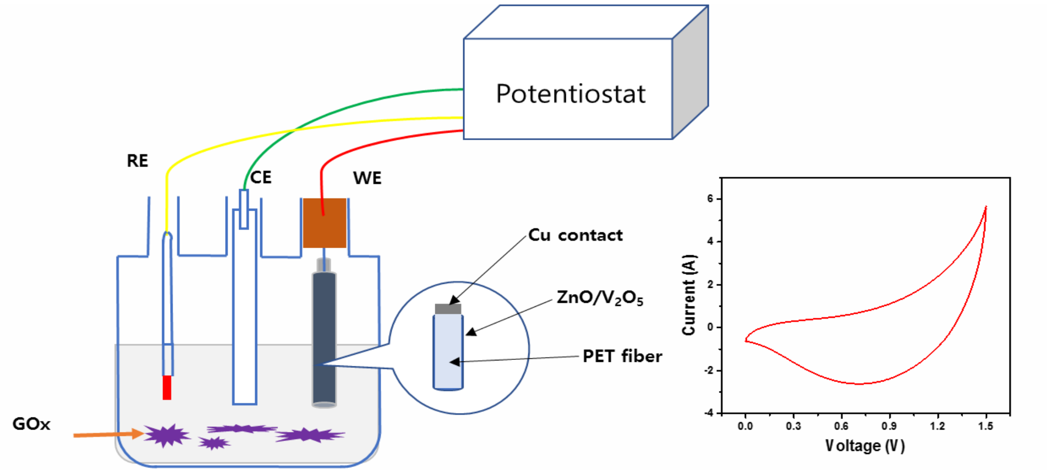

Scheme 2: Schematic representation of the electrochemical sensor testing. RE, CE and WE represent reference electrode, counter electrode and working electrode respectively

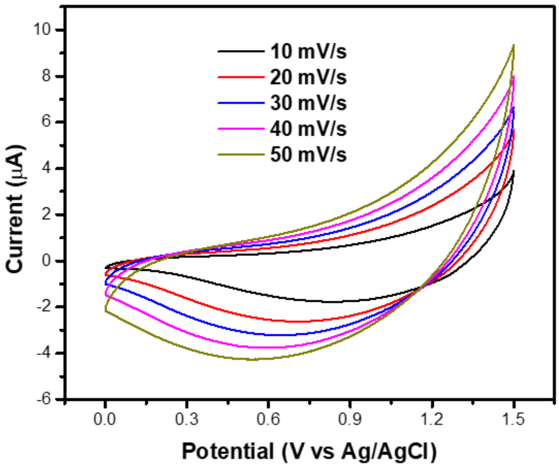

Figure S6. CV profiles for GOx in the absence of ZnO or ZnO/V_2O_5 (blank solution) at different scan rates.

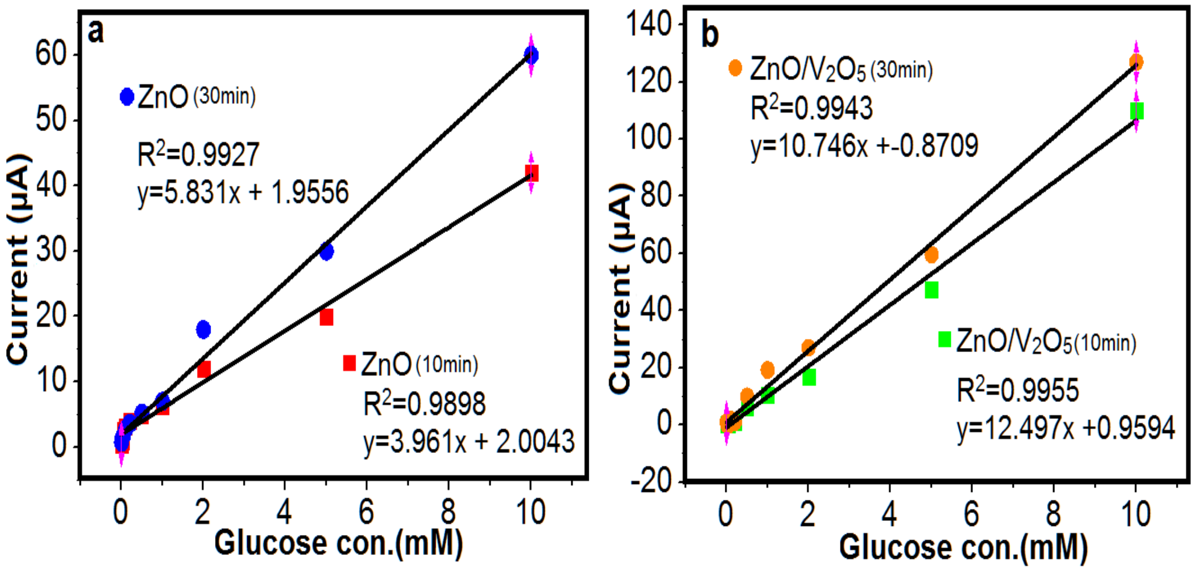

Figure S7. Calibration plot of ZnO and ZnO/V_2O_5 glucose sensors at +0.8 V with a straight line representing the linear fit for (a) 10 mins and (b) 30 mins ZnO seed layer deposition times on PET substrates

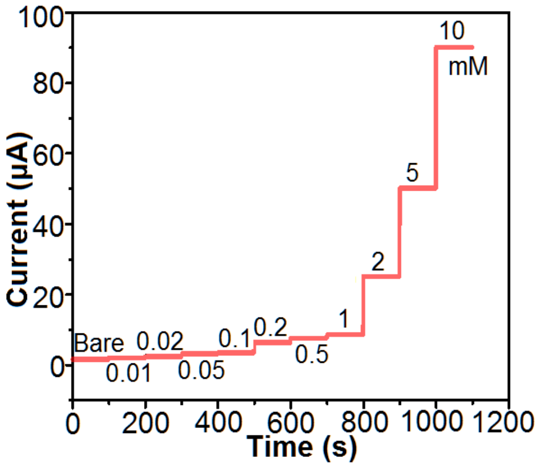

Figure S8. Current-time response monitoring according to increasing glucose concentration towards ZnO electrode

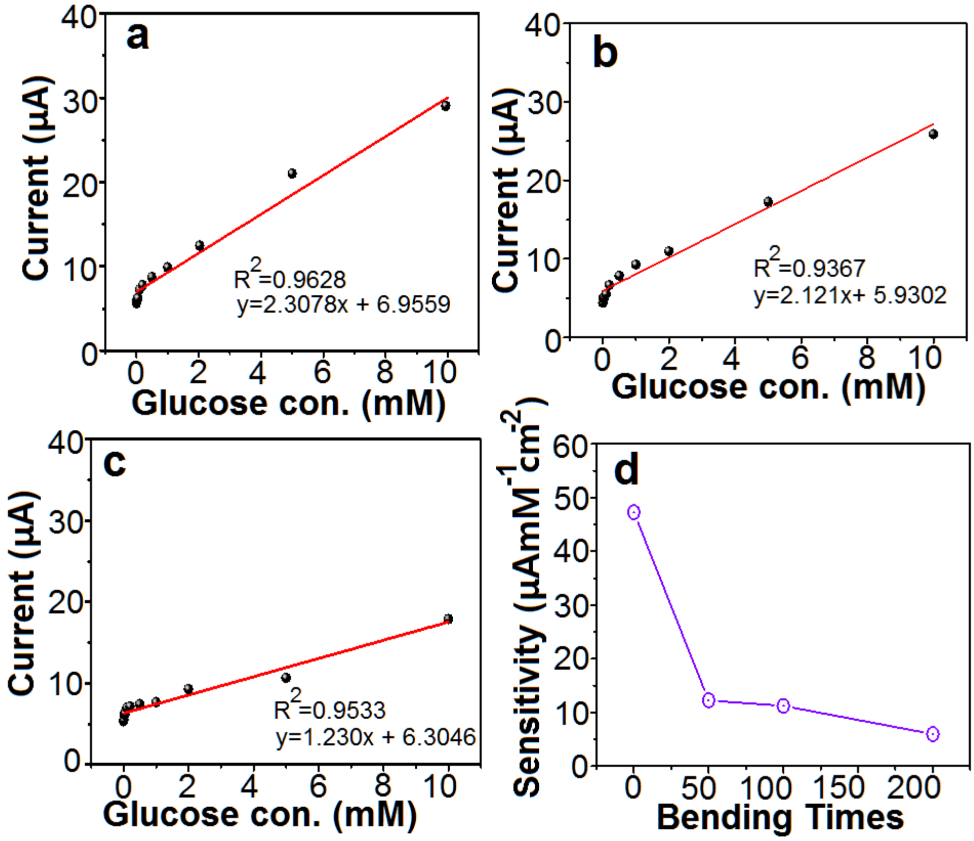

Figure S9. Calibration plot for ZnO/V_2O_5 glucose sensing at +1.0 V with a straight line representing the linear fit for ZnO seed layer deposited on PET for 20 min after (a) 50 cycles (b) 100 cycles (c) 200 cycles (d) Variation of calculated sensitivity after various bending cycles

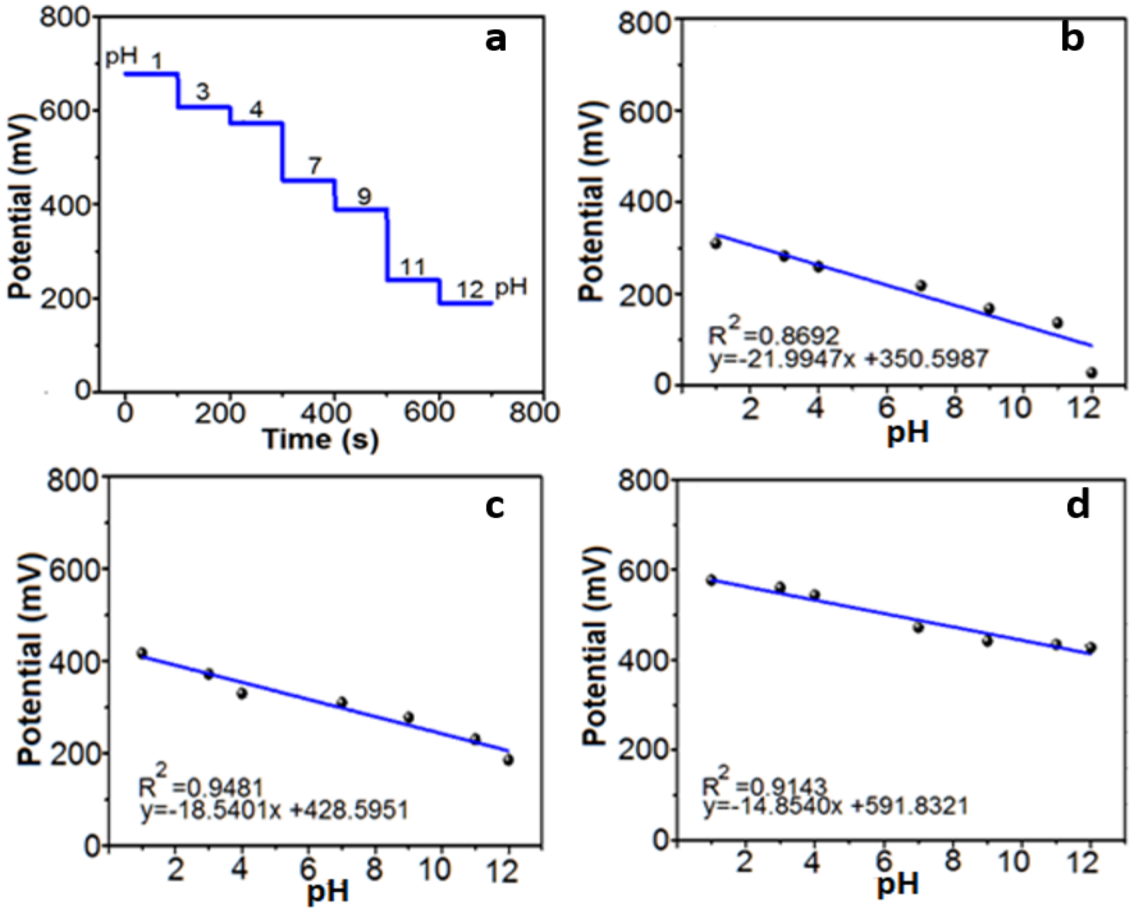

Figure S10. (a) Potential-time response obtained on increasing the pH of NaOH/HCl electrolyte for ZnO electrode. Calibration plot for ZnO/V_2O_5 pH sensors with a straight line representing the linear fit after (a) 50 cycles (b) 100 cycles (c) 200 cycles of repetitive bending.

Table S1. Sensitivity, limit of detection (LOD), and Limit of quantification (LOQ) of ZnO and ZnO/V_2O_5 glucose sensor on ZnO seed layer at different deposition times.

Electrodes	Deposition time, min	Sensitivity, $\mu AmM^{-1}cm^{-2}$	LOD, μM	LOQ, μM
ZnO	10	21.06	598	1990
	20	46.98	268	892
	30	30.67	392	1304
ZnO/V_2O_5	10	66.15	192	624
	20	72.06	174	582
	30	56.31	229	793
